# Plant phenological synchrony increases under rapid within-spring warming

**DOI:** 10.1038/srep25460

**Published:** 2016-05-05

**Authors:** Cong Wang, Yanhong Tang, Jin Chen

**Affiliations:** 1 State Key Laboratory of Earth Surface Processes and Resource Ecology, Beijing Normal University, Beijing, 100875, China; 2 College of Urban and Environmental Sciences, Peking University, Beijing, 100871, China

## Abstract

Phenological synchrony influences many ecological processes. Recent climate change has altered the synchrony of phenology, but little is known about the underlying mechanisms. Here using *in situ* phenological records from Europe, we found that the standard deviation (SD, as a measure of synchrony) of first leafing day (FLD) and the SD of first flowering day (FFD) among local plants were significantly smaller in the years and/or in the regions with a more rapid within-spring warming speed (WWS, the linear slope of the daily mean temperature against the days during spring, in ^o^C/day) with correlation coefficients of −0.75 and −0.48 for FLD and −0.55 and −0.23 for FFD. We further found that the SDs of temperature sensitivity of local plants were smaller under the rapid WWS conditions with correlation coefficients of −0.46 and −0.33 for FLD and FFD respectively. This study provides the first evidence that the within-season rate of change of the temperature but not the magnitude determines plant phenological synchrony. It implies that temporally, the asymmetric seasonal climatic warming may decrease the synchrony via increasing WWS, especially in arctic regions; spatially, plants in coastal and low latitude areas with low WWS would have more diverse spring phenological traits.

Phenological synchrony, which is referred to here as the temporal convergence of phenological events, can occur within individuals, among individuals or among species^1^,^2^,^3^. The synchrony of phenology has various ecological consequences for individual survival, species fitness and ecosystem stability^3^,^4^,^5^,^6^,^7^. For example, decreasing synchrony in flowering date can lead to assortative mating since early bloomers are more likely pollinated by other early plants, and late plants by late plants^8^. Besides, the synchrony among plants could augment the bee visitation rates by attracting more pollinators^9^ or decrease the rates by competing for pollinators^10^. There is increasing evidence that recent climate change has altered the phenological synchrony of various organisms^11^,^12^,^13^,^14^, but the underlying mechanisms are unclear, particularly in plants.

Both biotic and abiotic factors are expected to be involved in the phenological synchrony of plant^15^,^16^,^17^,^18^. Biotic factors, such as pollinator phenology and activity, may result in either high flowering synchrony^9^ or asynchrony^19^. Of the abiotic factors, temperature is the primary factor determining plant phenology^20^,^21^,^22^,^23^. Both the magnitude of the environmental temperature and the rate of the temperature change (speed) could affect phenological development, but to our knowledge, there is no evidence to date on whether or how the rate of change in environmental temperature affects plant phenological synchrony.

For a phenological event presumably determined only by temperature, there should be a range of environmental temperatures necessary or suitable for the event. Outside of this range, the plants would suffer from heat or cold stress. Flowering too early in the spring season may result in frost damage^24^,^25^, whereas flowering too late may decrease seed production as a result of high temperature stress^26^. The rate of seasonal change in environmental temperature, e.g., the speed of within-spring warming or within-autumn cooling, can differ between years and/or locations. A rapid within-spring warming period means a short period for any particular spring-season temperature assuming that the speed and direction of temperature change are monotonous and consistent (Fig. 1). We therefore hypothesized that under rapid spring warming conditions, plant phenology will have strong synchrony, thereby allowing a phenological event to align with the appropriate temperature within the short period available.

Here, using field observations of two spring events, the first leafing day (FLD) and the first flowering day (FFD) in Europe, we demonstrate that the synchrony of the timing of FLD and FFD among local plants are highly correlated with the within-spring warming speed (WWS, °C/day) both temporally and spatially. In addition, we show that the synchrony of phenological sensitivity of FLD and FFD to temperature as well are associated with WWS spatially.

## Results

### Temporal correlation

We firstly examined the degree of synchrony of FLD and FFD, in relation to the WWS temporally. The phenological data were selected from the Pan European Phenological Database (PEP725)^27^. The daily gridded climate data, with a spatial resolution of 0.25°, were obtained from the European Climate Assessment & Dataset project (E-OBS)^28^. The degree of phenological synchrony, i.e., the degree of timing-convergence, was assessed by the standard deviation (SD) of the FLD and FFD among all individual plants within an area coincident with the geographical grids defined in the climate data^1^. A low SD indicates a high degree of synchrony. The WWS for the FLD and FFD was calculated as the linear slope of the daily mean temperature against the days of the year during spring (see Method).

**Figure 2:**
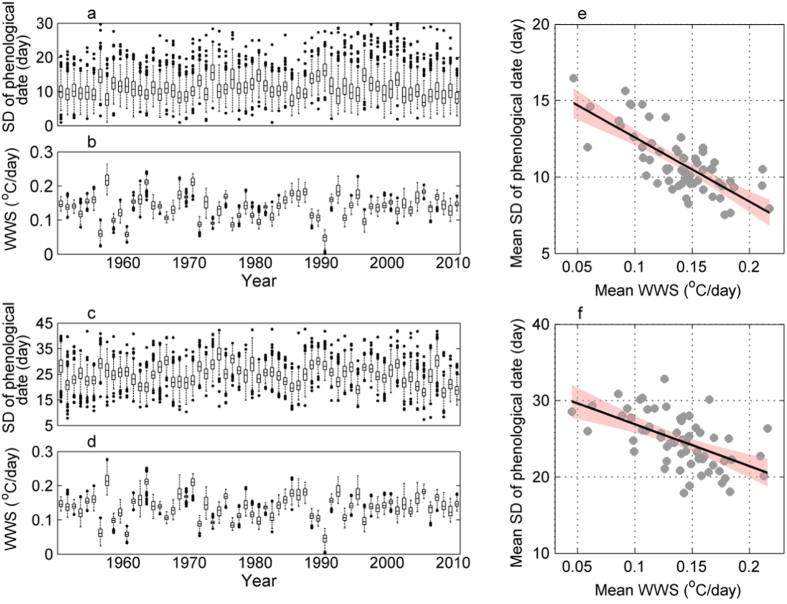
The standard deviation (SD) of spring phenological dates in relation to the within-spring warming speed (WWS, °C/day) during the period 1951–2011. (**a,c**) show the annual variations in the SD of the first leafing day (FLD) and the first flowering day (FFD). (**b,d**) show the WWS during spring for FLD and FFD. For each year, the SDs of the FLD or FFD were obtained for all the individual plants located within each grid. The bottom and the top of the box denote the 25th and 75th percentiles, respectively, and the line within the box represents the 50th percentile (the median). The whiskers extends to the maximum and the minimum SD excluding the outliers, which are the SD >(q3 + 1.5(q3 − q1)) or SD< (q1 − 1.5(q3 − q1)), where q1 and q3 are the 25th and 75th percentiles, respectively. (**e,f**) are scatterplots of the annual mean SD in relation to the annual mean WWS for the FLD (r = −0.75, P < 0.01) and FFD (r = −0.55, P < 0.01). The shaded region represents the 95% confidence interval of the regression line. All of the regressions have P-values  < 0.01.

During the period from 1951 to 2011, the average FLD and FFD for all individual plants in the PEP725 dataset advanced significantly, and the long-term advancement was closely associated with a distinct increase in the long-term temperature in the spring season (supplementary Figure 1). This is consistent with many previous claims that recent global warming has resulted in significant phenological shifts worldwide^11^,^13^,^29^,^30^,^31^. The SDs of the FLD and FFD among individual plants within each grid, however, exhibited large year-to-year fluctuations without a distinct long-term trend (Fig. 2a,c), indicating that the long-term temperature increase, i.e., the change in the temperature magnitude alone had no significant effect on the annual synchrony of the FLD or FFD.

We then examined the relationship between the annual SDs of the two spring events in relation to the within-spring warming speed. Visually, year-to-year fluctuations of WWS presented a rough inverse pattern with that of SDs (Fig. 2b,d). Decrease in SD was associated with increase in WWS. In addition, a simple linear regression analysis showed that the annual SDs of the FLD and FFD were both significantly higher in the years with lower annual WWSs (Fig. 2e,f). To reduce the bias caused by a small number of observational data in a grid, we also conducted the regression analysis after excluding grids containing fewer than 10 individuals and the results were consistent (supplementary Figure 2). Further partial correlation analysis confirmed that the annual SDs showed no significant partial correlation with the annual averaged spring temperature and total spring precipitation (Supplementary Figure 3). These results indicate that the rate of temperature increase but not the magnitude of temperature within the spring season is probably responsible for the annual fluctuation in the synchrony of the spring phenological events.

### Spatial correlation

**Figure 3:**
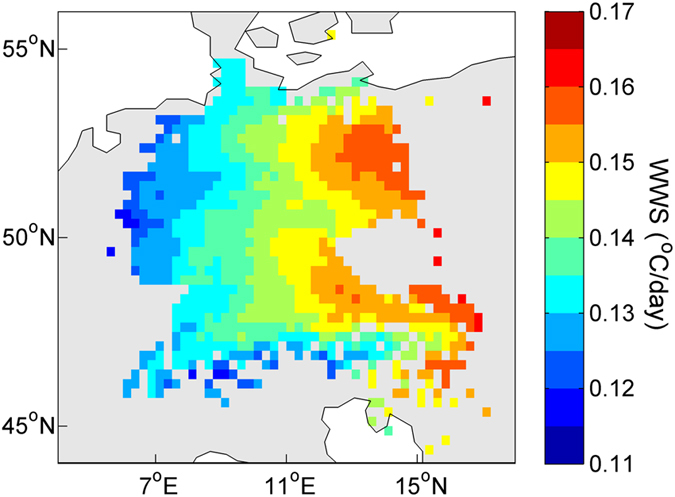
The geographical pattern of the within-spring warming speed (WWS, °C/day). The map was created using MATLAB 8.0 (http://cn.mathworks.com/).

**Figure 4:**
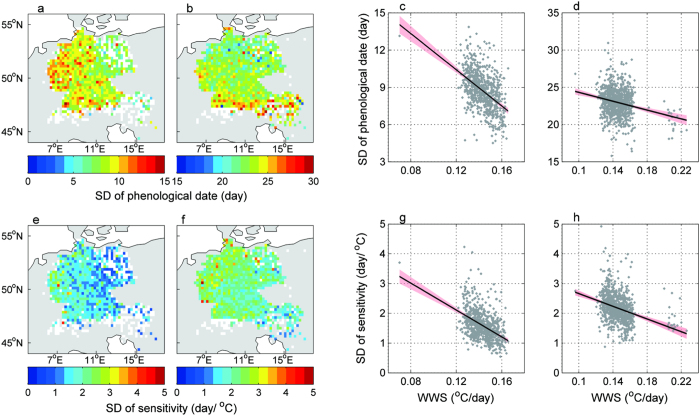
The spatial patterns of the standard deviation (SD) of spring phenological dates, the temperature sensitivity, and their association with the within-spring warming speed (WWS, °C/day). (**a**,**b**) show the SDs of the FLD and FFD; (**e**,**f**) show the SDs of the temperature sensitivity of the FLD and FFD, respectively. Each coloured point indicates the SD of the multi-year averaged phenological dates or their temperature sensitivity for all plants within a grid. The scatterplots show the SDs in relation to the WWS, with each data point denoting one grid. c and d show the SDs of the FLD and FFD associated with their WWS; (**g**,**h**) show the SDs of the FLD and FFD temperature sensitivity associated with their WWS, respectively. Grids with individual plant numbers <=10 are indicated by white colour and were not included in the correlation analysis. The line is the linear regression line, and the shaded region represents the 95% confidence interval. All of the regressions have P-values < 0.01. (**a,b,e,f**) were created using MATLAB 8.0 (http://cn.mathworks.com/).

The within-spring warming rate is also expected to change spatially. The WWS calculated from the multi-year mean daily temperature for each geographical grid was higher in inland areas than in coastal areas (Fig. 3). We investigated whether spatial changes in the WWS affected the synchrony of local plants. The results showed that the SDs of the multi-year mean FLD and FFD were lower in the inland grids than in the coastal grids, especially for the FLD (Fig. 4a,b). As a result, the SDs decreased significantly with the increase of the local WWS, with a correlation coefficient of −0.48 for the FLD and −0.23 for the FFD (Fig. 4c,d). Moreover, this correlation pattern was consistent for both individual plants grouped by each species and for species averages per grid (supplementary Figures 4 and 5).

Some factors may affect the correlations between the SDs and WWS. To evaluate whether any of the data processes introduced bias into the results obtained above, we performed the following procedures. First, to reduce the bias caused by a small number of observational data in a grid, we excluded grids containing fewer than 20 individuals (supplementary Figure 6). Second, to cope better with the phenological data, we examined synchrony by deeming each phenology station as the minimum unit instead of using the grid (supplementary Figure 7). Third, we examined the interquartile range (i.e., the upper quartile minus the lower quartile) instead of the SD to quantify synchrony (supplementary Figure 8). After the above procedures, we found that all of the results were consistent with our original findings. Finally, the results were also consistent with the initial results after controlling for other potential factors including the altitudinal variance, the spatial aggregation of the phenology stations, species diversity of the collected data within a grid as well as the mean spring temperature and total spring precipitation (supplementary method, supplementary Figure 9).

### Temperature sensitivity

The phenological trait, temperature sensitivity which characters the response of phenology to temperature, receives a lot of concerns in the context of global warming^18^,^19^,^26^. The synchrony of the temperature sensitivity as well has important ecological and evolutionary consequences^27^. We thus further examined whether the synchrony of the temperature sensitivity is also correlated to the within-spring warming speed. Herein, the temperature sensitivity was evaluated as a linear slope of the event dates with respect to an effective temperature (see method, days/°C). We conducted all the analysis performed above and found that in the areas with a slower WWS, the FLD and FFD show larger SDs of temperature sensitivity among individual plants, among individual plants grouped by each species or for species averages per grid (Fig. 4g,h; supplementary Figures 4–10). It is in line with the synchrony of the timing of phenological events.

## Discussion

Phenological synchrony among plants has long been a focus of ecologists and plant physiologists^1^,^3^,^4^,^28^. We find that temporally the degrees of synchrony in the first leaf unfolding day and the first flowering day among local plant individuals are highly dependent on the within-spring warming speed but not the magnitude of spring tempertaure. A previous research however showed that the synchrony of spring events was related with monthly mean temperature^32^. This difference may arise from the difference of the spatial scales in calculating the synchrony. A national standard deviation were calculated to represent synchrony on regional level in Menzel *et al.*^32^. While in the current study, the synchrony is defined at the grid or site level. On the basis of this finding, we assume that the rate of temperature increase within each spring season may act as a signal triggering the synchrony among individuals of different species. Physiologically, plants are able to sense either the magnitude of the temperature itself or the rate of temperate change^33^. Abrupt and gradual changes in temperature of the same magnitude may have different physiological consequences^33^,^34^,^35^. However, there is currently no evidence from either physiological or ecological studies to elucidate how the rate of temperature change induces yearly differences in phenological synchrony in plants.

The plant phenological traits are important determinants of fitness and the results of long-term adaptation to the local environment^36^,^37^. The close spatial correlation between the SDs of the multi-year mean phenological date or temperature sensitivity and the WWS among different grids indicates that the local rate of temperature increase within the spring season has strongly affected the adaption of the synchrony of the spring phenology. The potential role of the rate of temperature increase in influencing the adaption of phenological traits has been suggested in several previous literatures. B. VEEN (1954) hypothesised that early flushing trees would be selected out because of frequently frost damage if the climate has a gradually increasing mean temperature in spring^38^. A later research supported his hypothesis by growing seedlings from different locations under a uniform environment and finding that sources from north and inland area where daily mean temperature increase rapidly usually flushed early^39^. A recent research using satellite based vegetation index demonstrated that plants in areas with rapid within-spring warming were more sensitive to temperature in the Northern Hemisphere^40^. These studies jointly with the current research call for the caution that the within-season rate of change of the environmental temperature should be involved in the investigation of phenological adaption to local environment.

In temperate and boreal regions, temperature is usually the dominant factor determining the plant phenology^21^,^41^. However, in tropical and arid regions, the seasonal pattern of moisture availability may play more important role in regulating plant phenology^42^,^43^,^44^,^45^. Accordingly, the importance of the change rate of other environmental factors should be emphasized in these regions. Domínguez & Dirzo^42^ conducted two experiment designs, one mimicked a sudden increase in soil humidity and the other simulated a gradual one. They found a high synchrony of the FFD when moisture increase rapidly which is consistent with our speculation. However, to date there is still very little knowledge available for exploring the relation between the environment change rate and phenological synchronization. Further studies are needed.

The implications of our findings are multiple. Most importantly, the current study greatly improves our ability to predict the phenological synchrony both temporally and spatially. Recent climate change and climate projections all indicate a faster warming in the winter than in the summer, particularly in arctic regions^46^. This should result in a smaller range of variation in seasonal temperature in the spring and thus slower within-spring warming speed. The implication is that future climate change may increase phenological divergence if we assume that the phenological response to temperature change remains consistent. Spatially, low latitudes and coastal areas usually have gradual temperature increase in spring^40^. We thus predict that plants in these areas would present high levels of diversity in the phenological traits. We also analyzed the synchrony of temperature sensitivity in this research. The synchrony in sensitivity indicates the diversity in plants response to temperature. It could determine the species’ or communities’ ability to buffer and adapt to the changing climate^47^. In addition, this diversity can complicate efforts to predict response to climate change. On the basis of our result, when forecast future phenology pattern on large scales such as using satellite images, researchers should be aware that the estimates are more reliable in areas with high WWS because the responses of the plants are more synchronous in theses grids. Accordingly, incorporating this rate of change in analyses could help predict better plant phenology.

## Method

### Climate data

We used the E-OBS daily gridded dataset, with a spatial resolution of 0.25° for temperature and precipitation (http://www.ecad.eu). The E-OBS was derived from the interpolation of data from over 2,000 stations throughout Europe from ECA&D (European Climate Assessment and Data) and some additional data sets^28^. A prior study showed a very strong correlation between this dataset and the high-resolution regional gridded datasets in Europe^48^.

### Phenological data

The first leaf unfolding date (FLD) and the first flowering date (FFD) were obtained from the Pan European Phenology Database (PEP725; http://www.pep725.eu). In the dataset, there are 9 million records for 139 plants and 33 growth stages over approximately 20,000 locations across Europe (mainly in Germany, Austria and Switzerland) starting in the year 1868. These locations cover a region with a mean annual temperature ranging from 5 to 12 °C and an annual precipitation ranging from 550 to 1200 mm^49^. The dataset has been widely used in plant phenological studies^37^,^49^.

Subsets of records from PEP725 were selected for temporal and spatial analysis to maximize the data set size and conduct robust analyses. To select the data for temporal analysis, we (1) excluded data from before 1951 because of limited record numbers or/and overly spatially distributed records, (2) focused on individuals with a multi-year mean phenological date from 60 DOY to 150 DOY, and (3) focused on grids containing at least 3 records for any year during the study period (1951–2011). As a result, a total of approximately 20,000 time series within 3,000 observation stations over 13 plant species were included for the FLD analysis, and approximately 56,000 time series within 3,500 observation stations over 35 plant species were included for the FFD analysis.

The subset of records for spatial analysis was selected by performing steps (1) and (2) as with the temporal subset and (3) focusing on individuals with a time series longer than 15 years. As a result, a total of approximately 23,000 time series from 4,300 observation stations covering 25 plant species were included for the FLD analysis, and approximately 52,000 time series from 4,600 observation stations covering 39 plant species were included for the FFD analysis.

### Calculating the temperature sensitivity of spring phenology

The temperature sensitivity for a particular phenological event can be quantified as a linear slope of the event dates with respect to an effective temperature (days/°C)^21^,^40^,^50^. The effective temperature is often derived from the mean temperature of a period before the phenological event^20^,^40^. To obtain the effective temperature, we conducted a stepwise regression using the FLD and FFD as dependent variables against the independent variable, i.e., the monthly mean temperature, for each month from November of the previous year to June of the current year. We then performed a stepwise regression for each individual plant. The effective-temperature month was selected by the model with the input P-value of 0.05 and the output P-value of 0.1. To find the most effective period for a phenological event, we then obtained the percentage of the total number of individuals with the effective temperature out of the total number of individuals for each month:


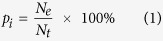


where *p*_i_ is the percentage for the month i, N_t_ is the total number of individual plants, and N_e_ is the number of individual plants for which the temperature of month i is effective. As a result, the effective temperature of the FLD for most individuals occurred in March and April, and that of the FFD for most individuals occurred in February, March and April (supplementary Figure 11). Within the months spanning the effective temperature, the majority of individuals, 98.4% for the FLD and 98.7% for the FFD, had a negative linear correlation with the monthly mean temperature, with 80.2% and 85.0% of the individuals yielding a P-value <0.05 for the FLD and FFD, respectively. We therefore used the above months for further analysis.

We also examined the results for temperature sensitivity for the following four cases: temperature sensitivity was recalculated after the effective temperature and phenological date were detrended, or the effective temperature was redefined as the mean temperature of the 30, 60 or 90 days before the multi-year mean phenological date for each individual. All the results showed results consistent with our original findings (supplementary Figure 10).

### Analysis

To examine the temporal correlation between the synchrony of phenological events and the within-spring warming speed (WWS), we obtained the standard deviation (SD) of the FLD and the FFD. For each year within each grid (0.25 × 0.25 degrees), the SDs were calculated for the phenological date of all plant individuals. The WWS was defined as the linear slope of the daily mean temperature against the days during spring (DOY 60–DOY 150). We then conducted a correlation analysis between the spatially averaged SDs and the WWS across years.

To conduct the spatial analysis, for each grid, the SDs were calculated for the yearly averaged phenological date and the temperature sensitivity of all plant individuals. The WWS was calculated using the yearly averaged daily mean temperature (1951–2011). We also calculated the SDs of all individuals by species and the species averages within each grid. Three species for the FLD and five species for the FFD with a large number of observations were used for the analysis at the species level (supplementary Table 1). We then conducted a correlation analysis between the SDs and the WWS across grids.

For the case of all of the individuals within a grid, we also performed a partial correlation analysis to exclude the potential influence of other factors (see supplementary method).

## Additional Information

**How to cite this article**: Wang, C. *et al.* Plant phenological synchrony increases under rapid within-spring warming. *Sci. Rep.* **6**, 25460; doi: 10.1038/srep25460 (2016).

## Supplementary Material

Supplementary Information

## Figures and Tables

**Figure 1:**
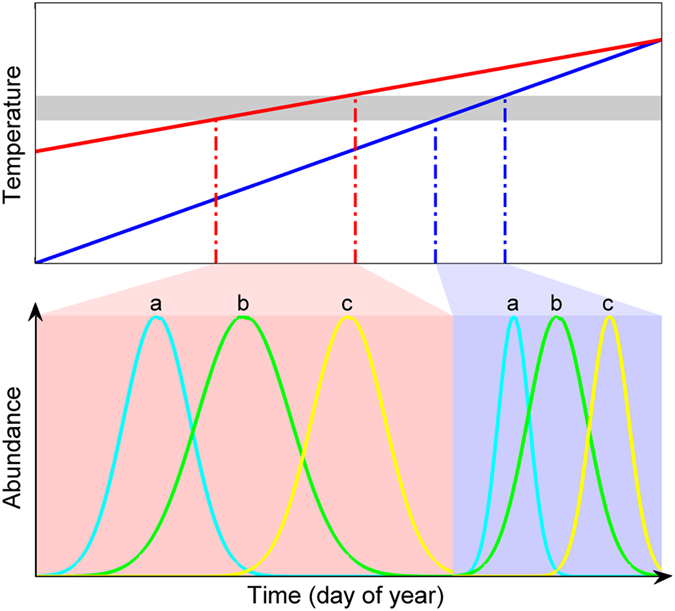
Conceptual diagram showing how the relative abundance of a phenological event is distributed temporally. The upper panel shows two typical time series for temperature elevation within the spring season. If a phenological event requires a range of necessary or suitable temperatures and that range does not change, a rapid temperature elevation would correspond to a short period, as indicated on the right in the bottom panel, and a slow temperature elevation would correspond to a long period. In the bottom panel, the letters a, b, c can represent three individuals or three species within a habitat or within a phenological period, such as the spring season.
