# Supplementary material for: Plant phenological synchrony increases under rapid within-spring warming
Source: Sci Rep. 2016 May 5;6:25460. doi: 10.1038/srep25460 (PMC4857096; doi:10.1038/srep25460)
Supplement: Supplementary Information [file srep25460-s1.pdf]

Plant phenological synchrony increases under rapid within-spring warming

Cong Wang, Yanhong Tang, Jin Chen

Supplementary Method

Following factors were controlled in the spatial partial correlation analysis.

Firstly, the altitudinal variation within a grid. We used the Global 30 Arc-Second Elevation Data Set (GTOPO30) with a grid spacing of 30 arc seconds developed by USGS (http://eros.usgs.gov) to obtain the standard deviation of the elevation values within a grid of E-OBS.

The degree of spatial aggregation of the phenology stations within a grid is another important factor since the phenological data in a grid may be collected from different phenological stations. We examined the degree of spatial aggregation of the stations using the following model:

$$J = 2\sqrt{n/A} \times \overline{r} \qquad \text{Supplementary Equation (1)}$$

Where n is the number of the stations, A is the area of a grid and $\bar{r}$ is the average distance of the nearest station. J is defined as zero if there is only one station in a grid.

Species diversity of the collected data within a grid was also examined using the Shannon's diversity index[1]:

$$H = -\sum_{i=1}^{s} p_i \ln p_i \qquad \text{Supplementary Equation (2)}$$

Where S is the number of the species, p_i is the proportion of ith species.

Finally, we noticed the different phenology between wild plants and cultivated plants. Since this issue can be considered as a special case of species diversity, we used the same definition with Supplementary Equation (2) to quantify the effects of wild plants and cultivated plants.

These effective factors along with the total spring precipitation (TSP) and the mean spring temperature (MST) were controlled in the partial correlation analysis.

Supplementary Reference

1 Keylock, C. Simpson diversity and the Shannon–Wiener index as special cases of a generalized entropy. *Oikos* **109**, 203-207 (2005).

Supplementary Figures

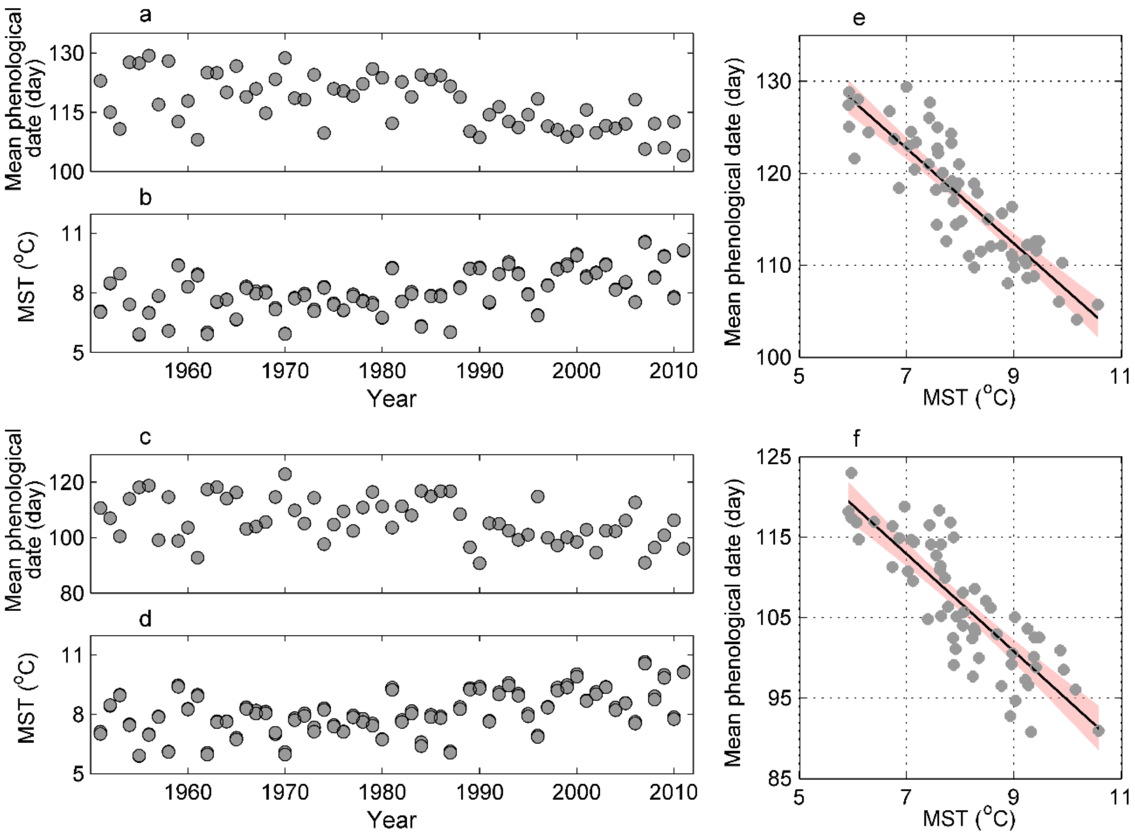

Figure 1 The mean spring phenological date in relation to the mean spring temperature (MST, ^{o}C) during the period 1951-2011. a and **c** show the annual variations in the mean of the first leafing day (FLD) and the first flowering day (FFD). **b** and **d** show the annual variantions of MST for FLD and FFD. **e** and **f** are scatterplots of yearly mean phenological dates in relation to the MST for FLD (r=-0.87, P<0.01) and FFD (r=-0.84, P<0.01). The shaded region represents the 95% confidence interval of the regression line. All of the regressions have P-values <0.01.

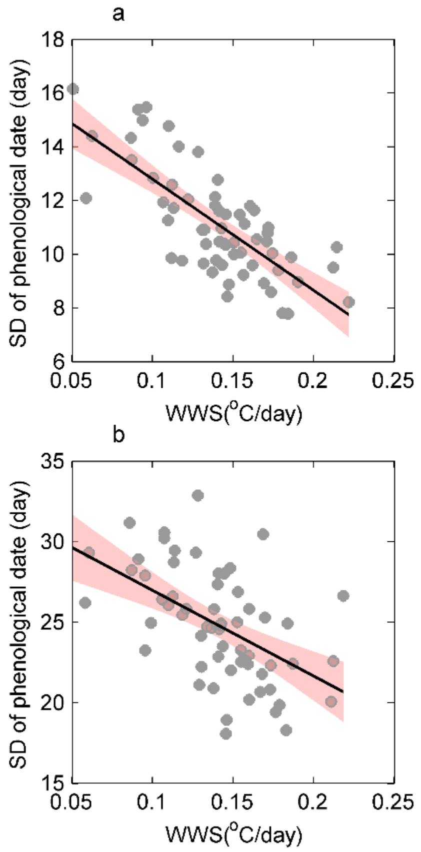

Figure 2 The standard deviation (SD) of spring phenological dates in relation to the within-spring warming speed (WWS, °C/day) during the period 1951-2011. a and **b** are scatterplots of the annual mean SD in relation to the annual mean WWS for the FLD (r=-0.75, P<0.01) and FFD (r=-0.54, P<0.01). The shaded region represents the 95% confidence interval of the regression line. All of the regressions have P-values <0.01. Here, only grids containing at least 10 records for any year during the study period (1951-2011) were involved.

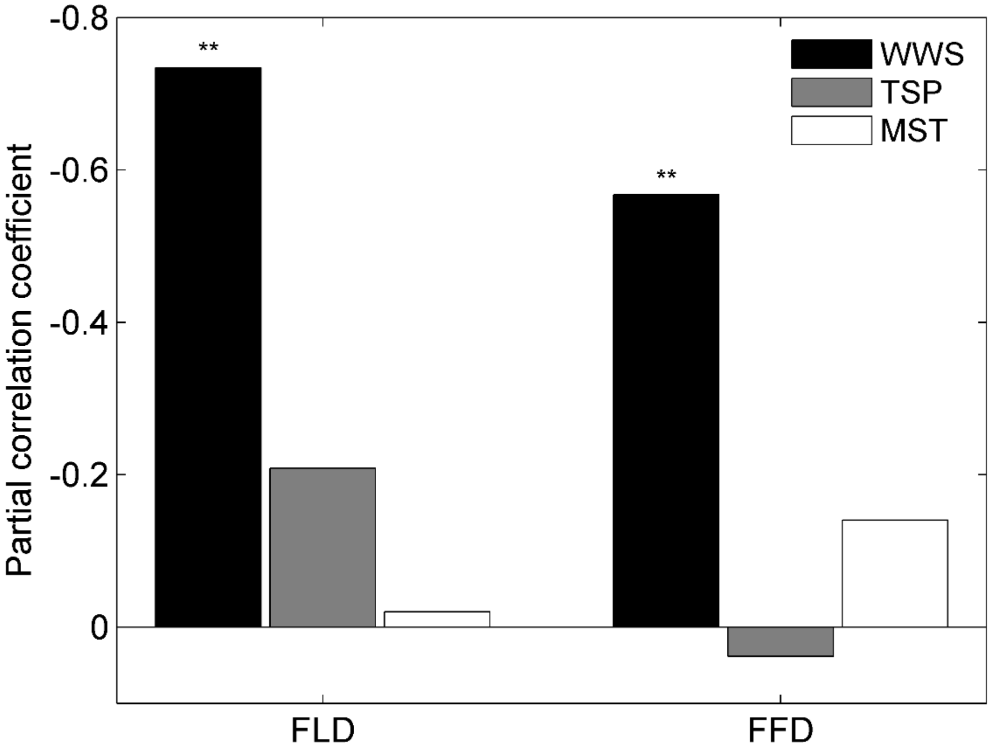

Figure 3 Temporal partial correlation between the standard deviation (SD) of the phenological date and the within-spring warming speed (WWS, ^{o}C/day), the total spring precipitation (TSP, mm) and the mean spring temperature (MST, ^{o}C) during the period 1951-2011. The partial correlation coefficient between SD and a climate factor was calculated after controlling the other two factors. ** represents P-value <0.01.

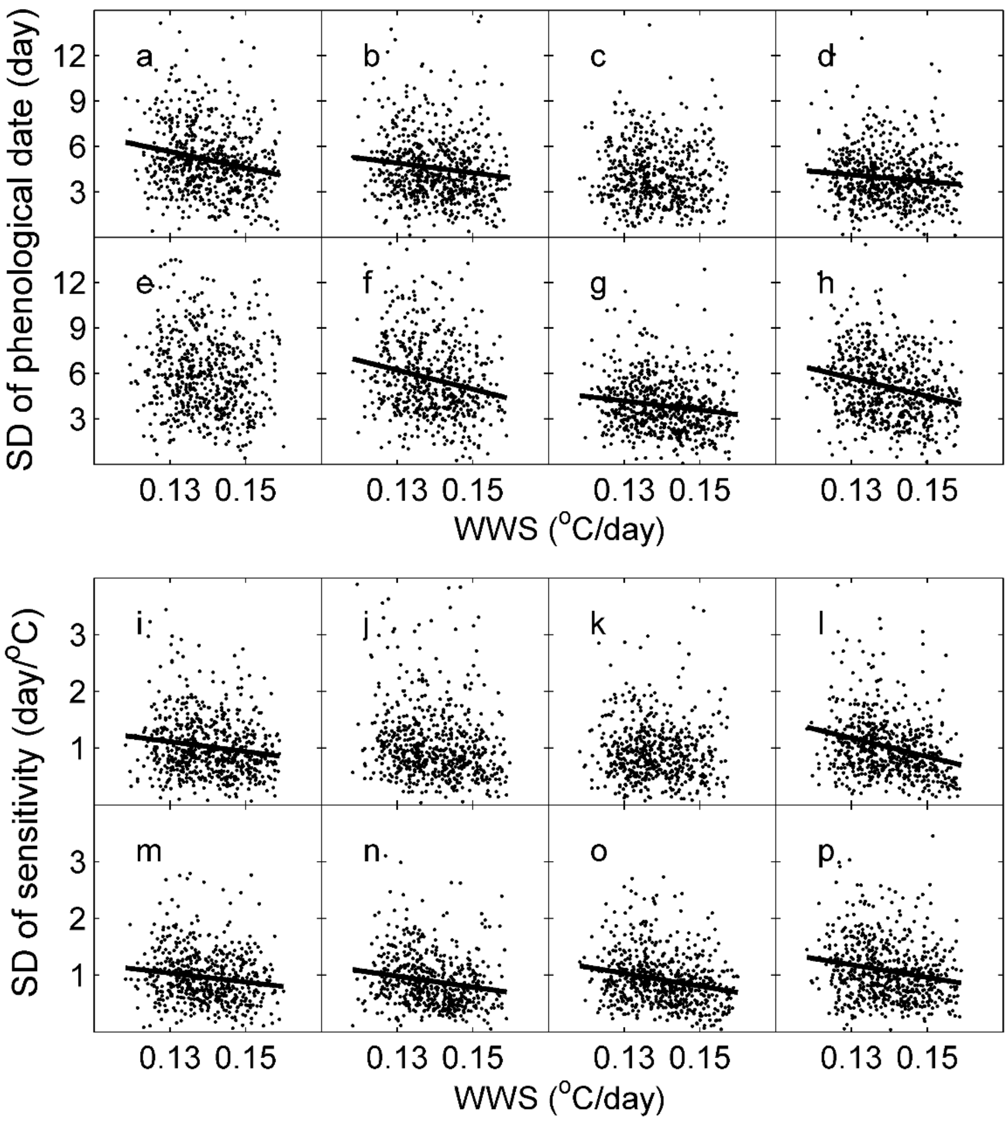

Figure 4 The relationship between the within-spring warming speed (WWS, °C/day) and the standard deviation (SD) of the phenological date and their temperature sensitivity, respectively, among the individuals for each species within a grid. The linear regression line is indicated if the correlation coefficient has P-value <0.01. **a** and **i** show FLD of *Aesculus.hippocastanum*; **b** and **j** show FLD of *Betula.ss*; **c** and **k** show FLD of *Fagus.ss* ; **d** and **l** show FFD of *Aesculus.hippocastanum*; **e** and **m** show FFD of *Tussilago. Farfara*; **f** and **n** show FFD of *Salix.ss;* **g** and **o** show FFD of *Syringa. Vulgaris*; **h** and **p** show FFD of *Taraxacum.officinale.*

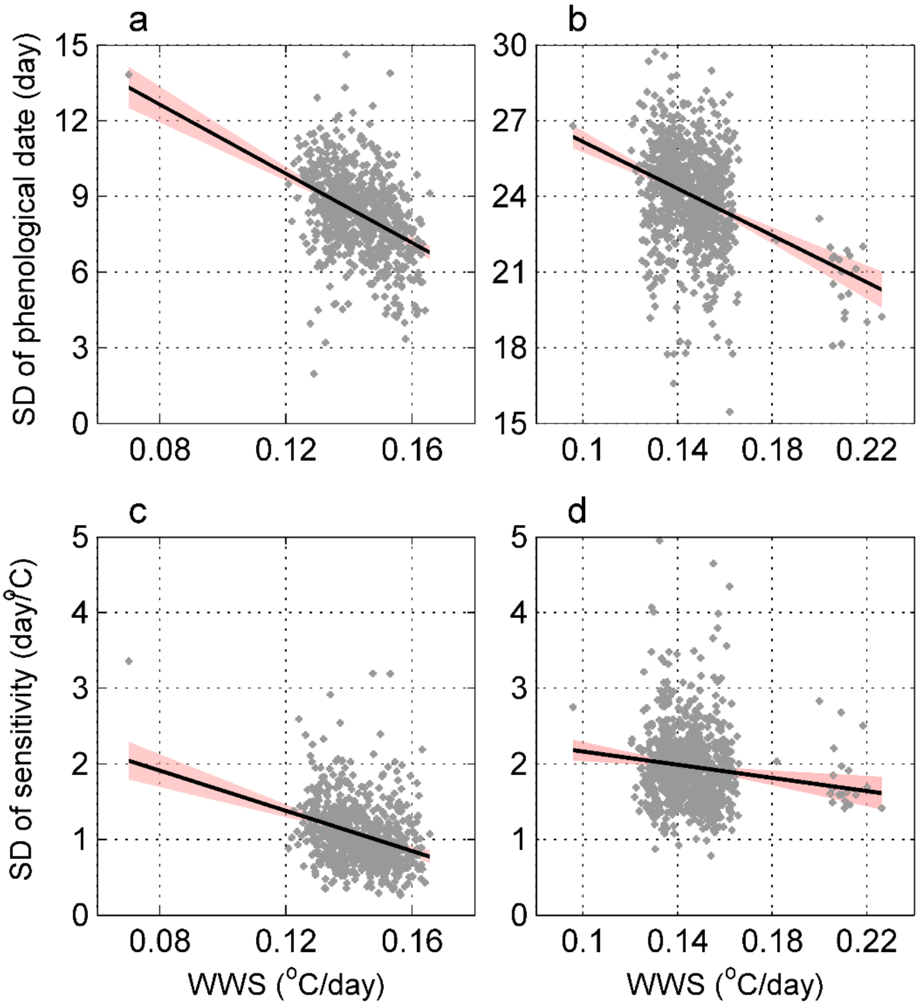

Figure 5 The relationship between the within-spring warming speed (WWS, °C/day) and the standard deviation (SD) of the phenologcial date and their temperature sensitivity, respectively, among the species within a grid. **a** and **c** show FLD; **b** and **d** show FFD. Only pixels contain more than 10 individuals are involved. The shaded region represents the 95% confidence interval of the regression line. All of the regressions have P-values <0.01. The correlation coefficients are -0.41, -0.33, -0.28, -0.11 for a, b, c, d respectively.

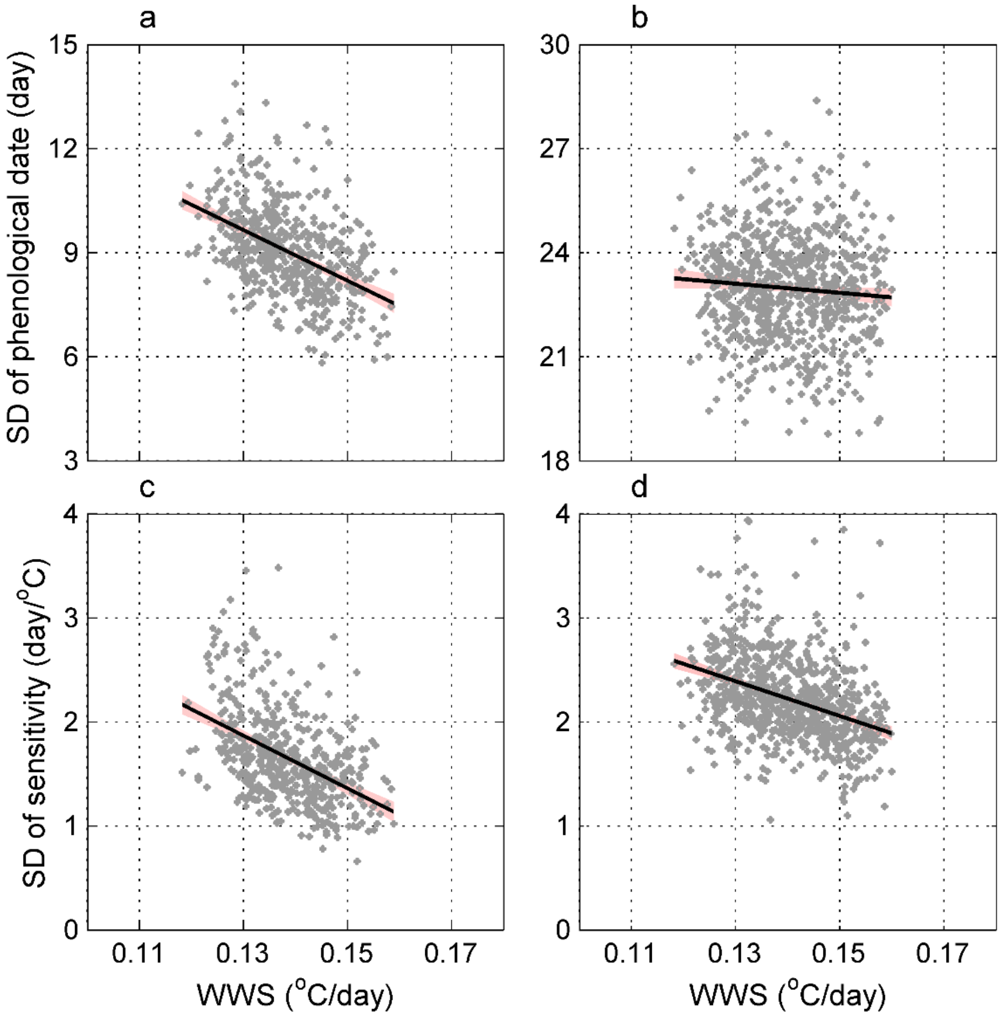

Figure 6 The relationship between the within-spring warming speed (WWS, °C/day) and the standard deviation (SD) of the phenological date and their temperature sensitivity, respectively, among all the individuals within a grid. a and **c** show FLD; **b** and **d** show FFD. Grids with individual plant numbers <20 are excluded. The shaded region represents the 95% confidence interval of the regression line. All of the regressions have P-values <0.01 except for b (P=0.03). The correlation coefficients are -0.46, -0.08, -0.45, -0.37 for a, b, c, d respectively.

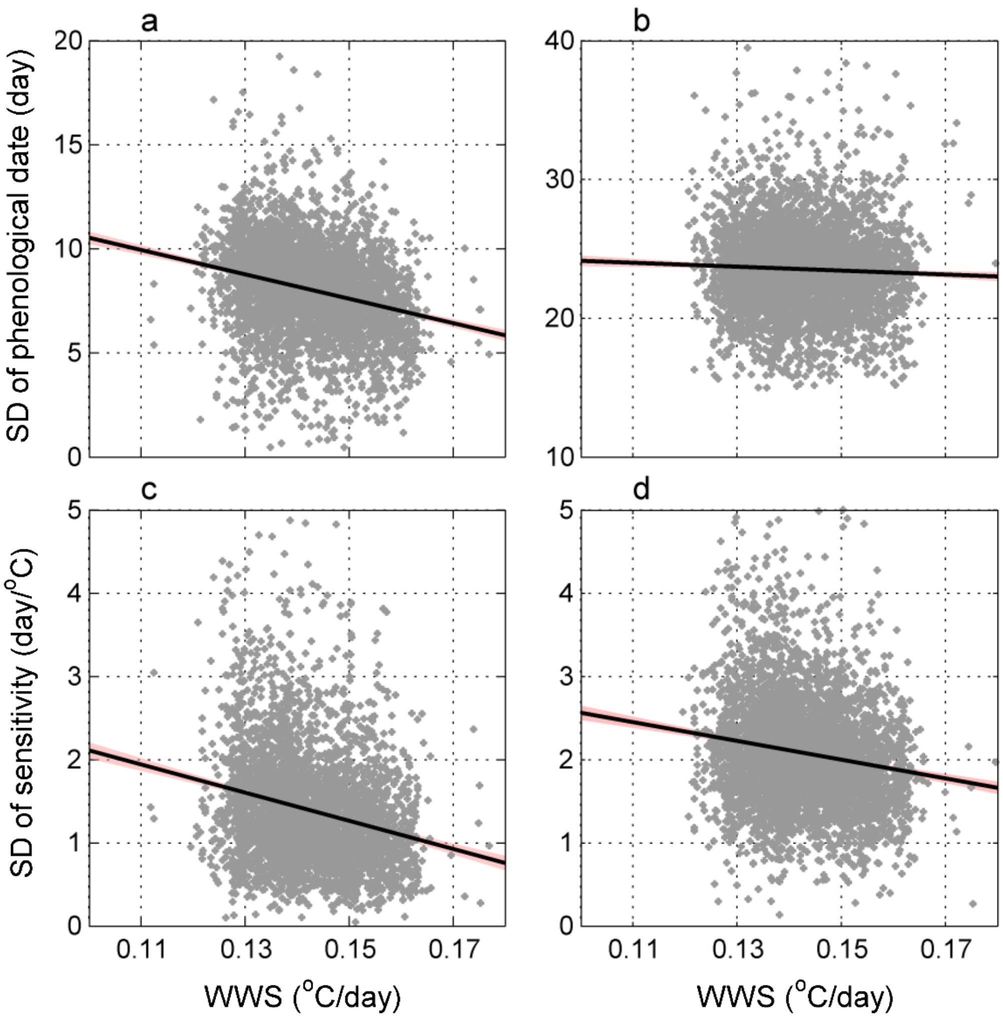

Figure 7 The relationship between the within-spring warming speed (WWS, °C/day) and the standard deviation (SD) of the phenologcial date and their temperature sensitivity, respectively, among all the individuals within a phenological station. a and **c** show FLD; **b** and **d** show FFD. The shaded region represents the 95% confidence interval of the regression line. All of the regressions have P-values <0.01. The correlation coefficients are -0.26, -0.05, -0.23, -0.17 for a, b, c, d respectively.

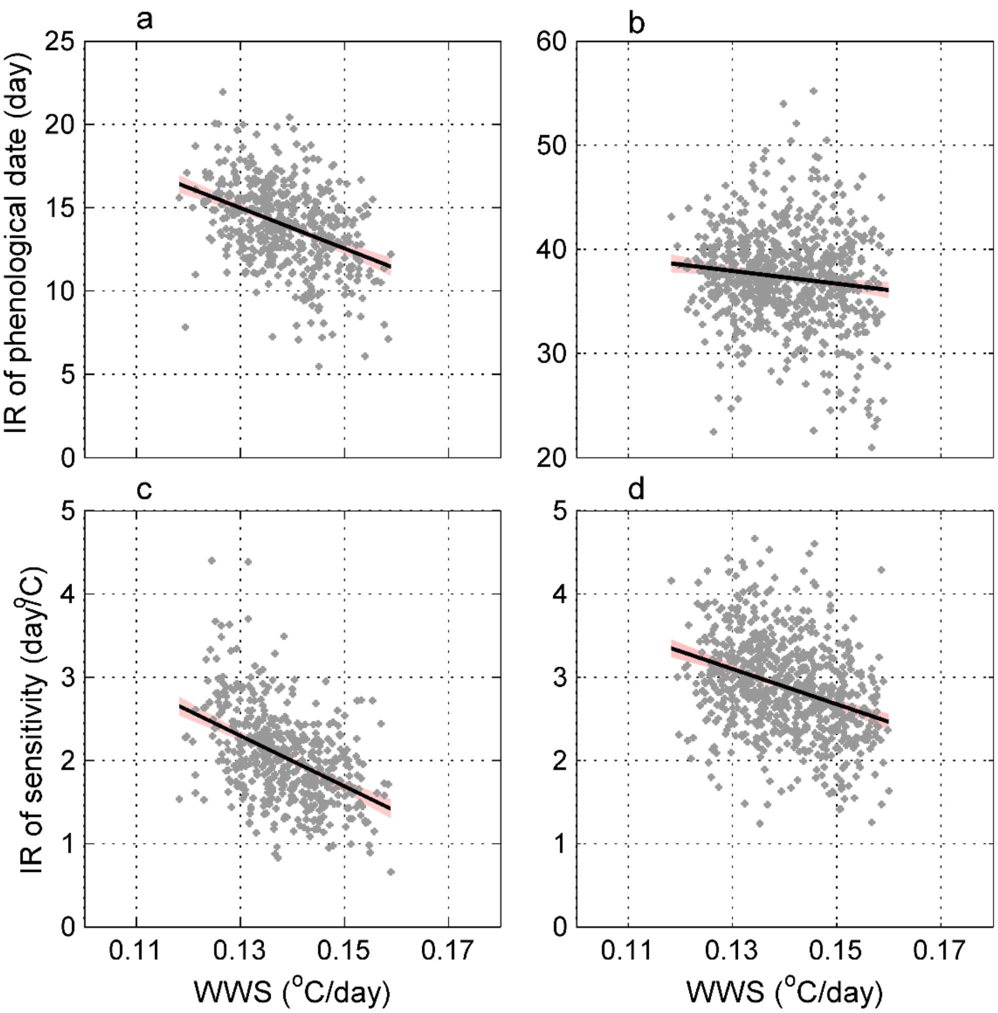

Figure 8 The relationship between the within-spring warming speed (WWS, °C/day) and the interquartile range (IR) of the phenological date and their temperature sensitivity, respectively, among all the individuals within a grid. a and **c** show FLD; **b** and **d** show FFD. Grids with individual plant numbers <20 are excluded. The shaded region represents the 95% confidence interval of the regression line. All of the regressions have P-values <0.01. The correlation coefficients are -0.40, -0.13, -0.46, -0.33 for a, b, c, d respectively.

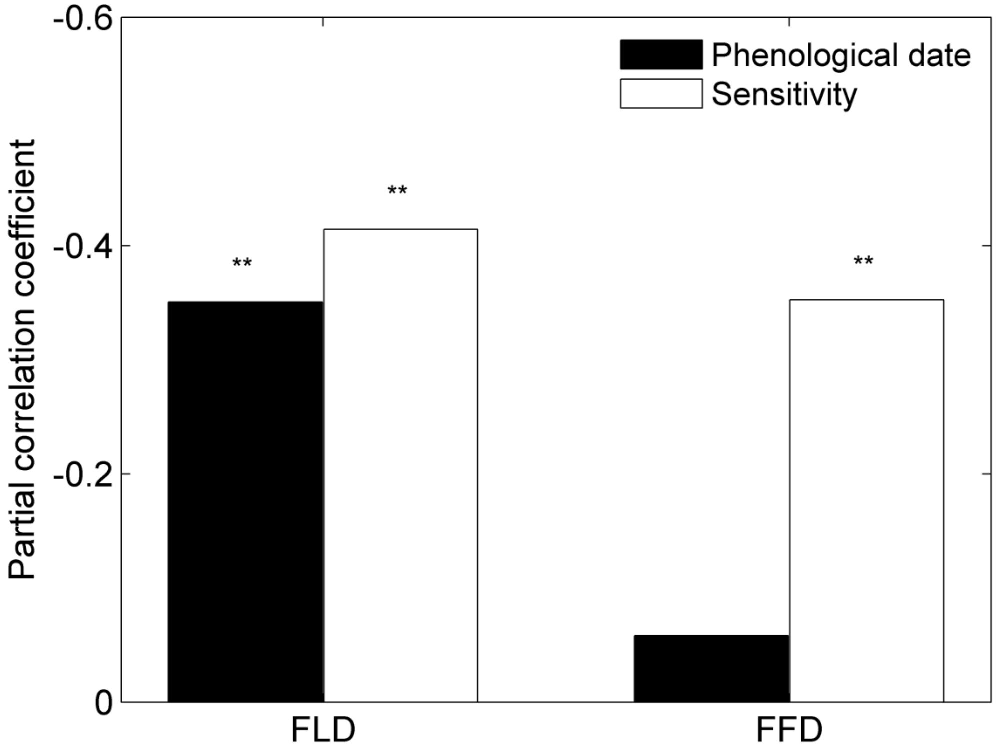

Figure 9 Partial correlation coefficient between the standard deviation of phenological date and temperature sensitivity among all the individuals within a grid and the within-spring warming speed (WWS, ^{o}C/day). The partial correlation coefficient was calculated after controlling the total spring precipitation, the mean spring temperature and the other potential factors. Only grids contain more than 10 individuals are involved. ** represents P-value <0.01.

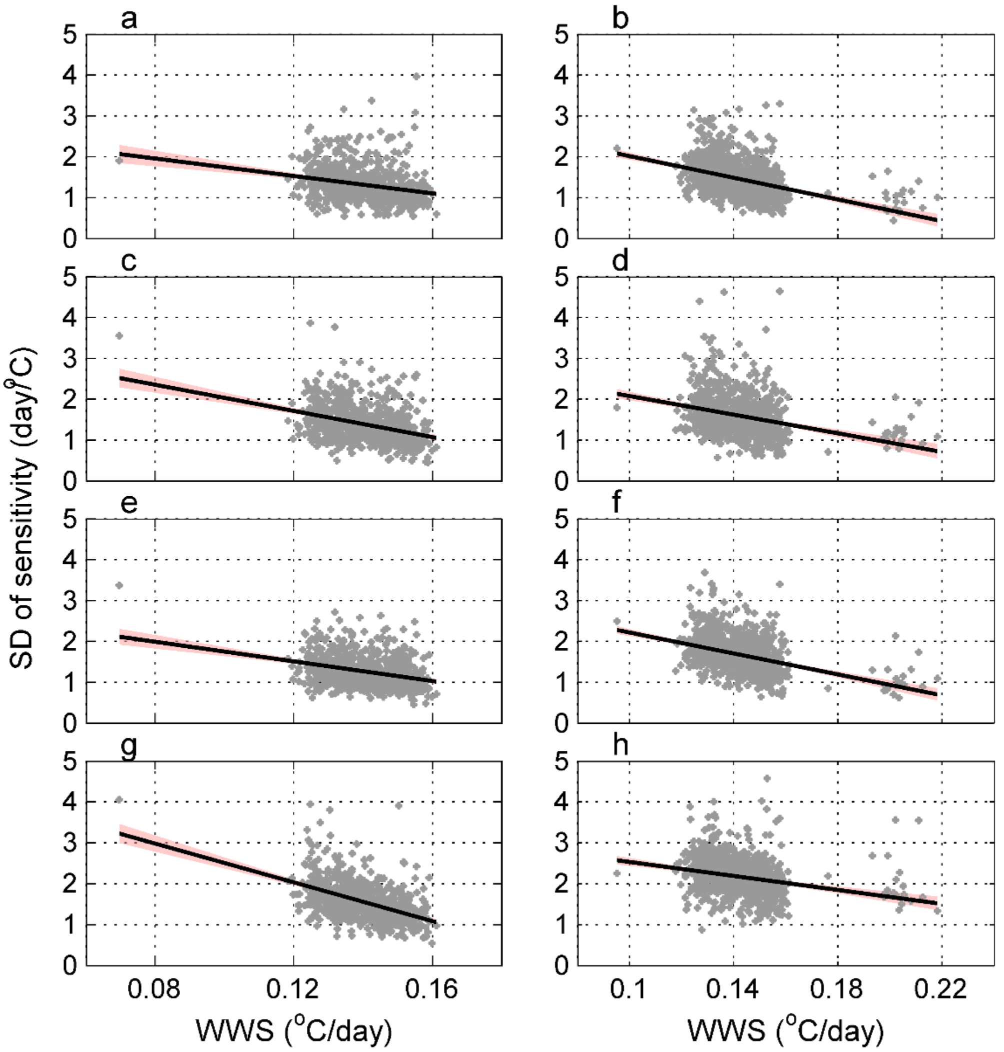

Figure 10 The relationship between the within-spring warming speed (WWS, °C/day) and the standard deviation (SD) of the temperature sensitivity among all the individuals within a grid. a, c, e and **g** show FLD**; b, d, f** and **h** show FFD. The effective temperature for (**a**, **b**), (**c**, **d**), (**e**, **f**) is the mean temperature of the 30, 60 or 90 days before the multi-year averaged phenological date for each individual. For (**g**, **h**), temperature sensitivity was calculated after the effective temperature and phenological date were detrended. Only grids contain more than 10 individuals are involved. The shaded region represents the 95% confidence interval of the regression line. All of the regressions have P-values <0.01. The correlation coefficients are -0.24, -0.40, -0.34, -0.30, -0.31, -0.40, -0.47, -0.25 for a, b, c, d, e, f, g, h respectively.

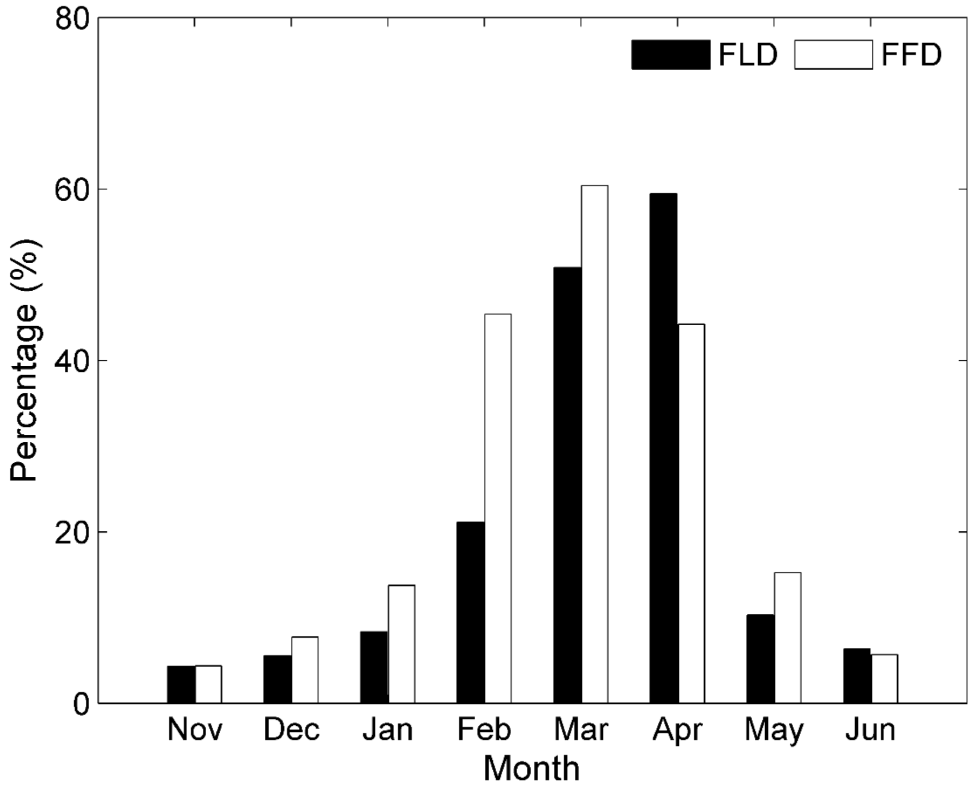

Figure 11 The percentages of first leaf day (FLD, black bar) and first flower day (FFD, white bar) of the individuals with effective temperature out of the total individuals for each month.

Supplementary Table

Table 1 **Species used for the calculation of synchrony at the species level.** Species name and number of pixels involved in the analysis are exhibited.

**phenology**	**Species name**	**Number of pixels**
FLD	Aesculus *hippocastanum* (A.*h*)	635
FLD	Betula (B.*ss*)	627
FLD	Fagus (F.*ss*)	542
FFD	Aesculus *hippocastanum (*A.*h)*	609
FFD	Tussilago *farfara (*T.*f)*	576
FFD	Salix (S.*ss*)	544
FFD	Syringa *vulgaris (*S.*v)*	633
FFD	Taraxacum *officinale (*T.*o)*	624
